# A discrete choice experiment with health professions trainees to improve the urban-rural health care access disparity in Appalachia: Study protocol

**DOI:** 10.1371/journal.pone.0316521

**Published:** 2025-01-13

**Authors:** Chris Gillette, Jan Ostermann, Sarah Garvick, Chris Everett, Dawn Caviness, Aylin A. Aguilar

**Affiliations:** 1 Department of PA Studies, Wake Forest University School of Medicine, Winston-Salem, North Carolina, United States of America; 2 Department of Epidemiology and Prevention, Wake Forest University School of Medicine, Winston-Salem, North Carolina, United States of America; 3 Department of Health Services Policy and Management, Arnold School of Public Health, University of South Carolina, Columbia, South Carolina, United States of America; 4 Department of Health Sciences Education, Medical College of Wisconsin, Milwaukee, Wisconsin, United States of America; 5 Cabarrus Family Medicine Residency Program, Atrium Health, Concord, North Carolina, United States of America; 6 Department of Social Sciences and Health Policy, Wake Forest University School of Medicine, Winston-Salem, NC, United States of America; PLOS: Public Library of Science, UNITED KINGDOM OF GREAT BRITAIN AND NORTHERN IRELAND

## Abstract

Globally, those who live in rural areas experience significant barriers to accessing health care due to a maldistribution of health care providers. Those who live in rural areas in the Appalachian region of the United States face one of the worst shortages of health care providers despite experiencing more complex health needs compared to Americans in more affluent, urban areas. Prior research has failed to identify effective solutions to narrow the provider maldistribution, despite it being a policy focus for decades. More work is needed to better understand the complex, multidimensional process in which health care providers select jobs and how job, community, and providers’ intrapersonal characteristics influence job selection. This paper is a protocol for a study aimed at identifying effective policies and incentives to improve recruitment of healthcare providers for their first job in rural Appalachia. We will use rigorous, theoretically grounded discrete choice experiment methodology (DCE) to accomplish the study’s objective. The main outcome will be the relative importance of alternative community and job characteristics for trainees’ choices of jobs in rural Appalachia. secondary outcomes of interest will be trade-offs that these trainees make when selecting a job, described in the form of marginal rates of substitution (mRS). Participants include medical residents and fellows, PA students and NP students in their final year of training. The choice context will be the recruitment of these trainees for their first job. Data will be analyzed using mixed logit analysis. Results from this DCE will improve our understanding of the job selection process for health care providers. The identification and prioritization of predictors of trainees’ rural job choices will allow for the development of policies and incentives that will enable policymakers and health care systems to recruit more providers to rural and underserved areas.

## Introduction

**Fig 1:**
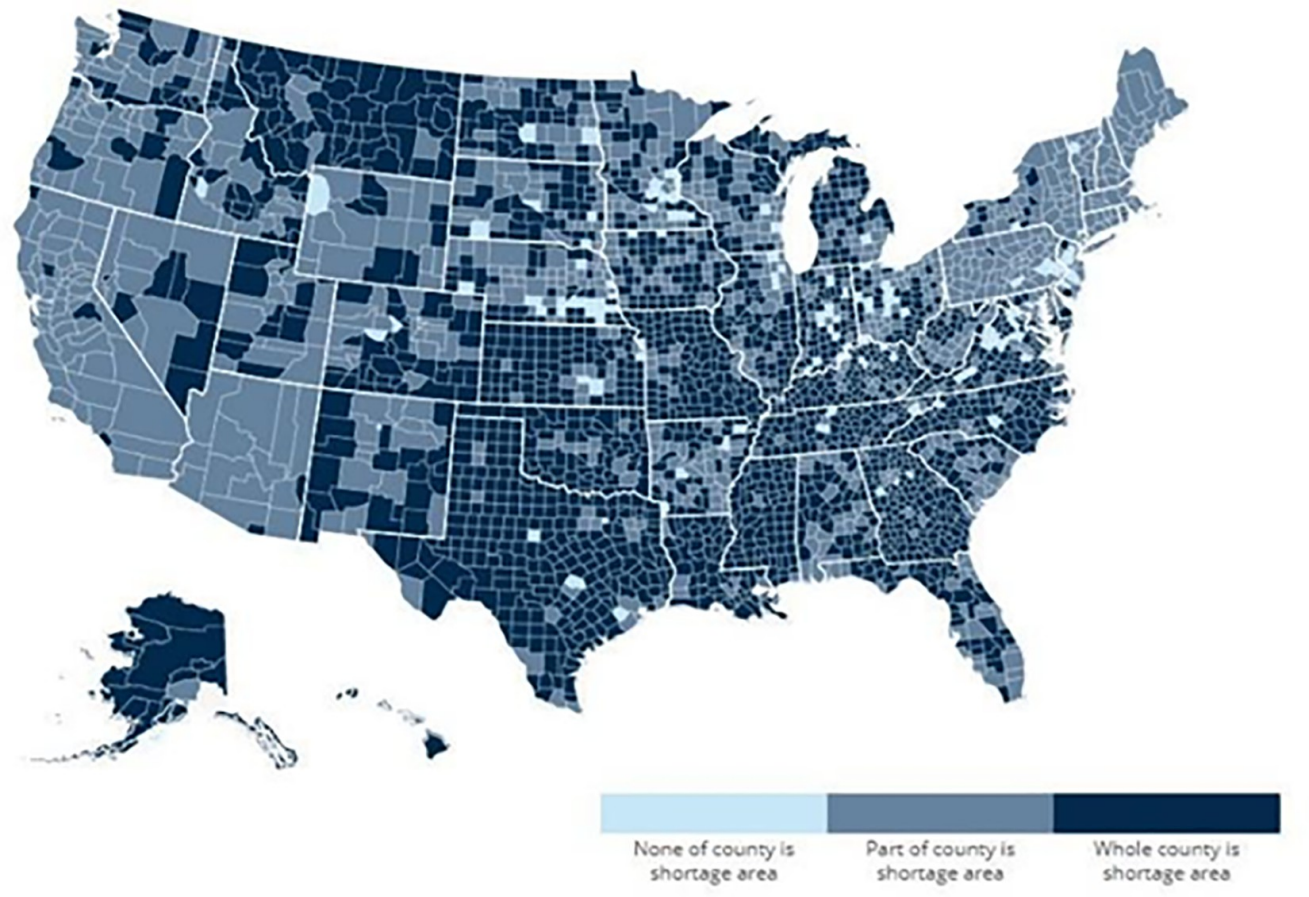
Health professional shortage areas, 2022.

About one in five Americans live in a rural area [1]. Compared to their urban counterparts, rural Americans are more likely to die early from the most common causes of death in the United States (US). Rural Americans are also more likely to forego needed health care because of being uninsured or underinsured [2]. There is a greater need for health care in rural areas, however, those who live in rural areas face inequities in accessing needed health care (Fig 1) [3]. The Appalachian region of the US is among the most affected areas regarding health inequities in the country [4]. While having a greater need for health care, Appalachia experiences some of the worst shortages of healthcare professionals in the US [5]. In fact, coupled with disparities in morbidity and mortality, health care access disparities in Appalachia have led to a 25% greater loss of potential life years than the rest of the US [6]. According to the Behavioral Model of Health Services Use, the *combination* of poor predisposing factors (e.g., being older in age than the rest of the nation) and lack of enabling factors (e.g., low availability of clinicians) contribute to widening health disparities for those who live in the rural US and especially for Appalachians compared to urban areas [7].

Despite decades of research efforts to identify the best ways to improve rural medical practice, the rural-urban health access disparity continues to worsen [8]. The most consistent factor so far associated with choosing to practice in a rural/underserved area is location of upbringing; health care professionals who were raised in a rural area are more likely to practice in a rural area after their medical training [8]. Decreasing numbers of Americans from rural backgrounds are choosing to go to medical school so other efforts are needed to improve rural access to care [9]. In response to these findings, medical schools have implemented pipeline programs for middle- and high-school adolescents from rural areas to get them acquainted with a medical career [8, 10, 11]. However, these strategies are heterogeneous in their approaches and as such, there is a dearth of literature on whether these programs are successful. When these programs have been studied, the outcome is a change in enrolled students’ demographics instead of identifying the actual effectiveness of the pipeline itself by tracking pipeline students’ trajectories [12].

Another intervention that medical schools and other health professions training programs have implemented to improve the rural-urban health access disparity are rural clinical rotations [8, 13]. A recent systematic review found that training programs located in rural areas have the most robust evidence for effectiveness in multivariable analyses; both qualitative and quantitative research methods show, however, that rural clinical rotations may be more dependent on the background of the trainee instead of the training program when choosing a job in a rural area [8, 13, 14].

The most popular policy implemented at the state and federal government level for recruiting medical trainees to rural areas are medical school loan repayment or forgiveness programs in exchange for a certain number of years of service in a rural/underserved community [15, 16]. Previous work has found that these programs may be enticing to some medical graduates to practice in a rural community while other research found that the physicians and other health professionals who used these programs would likely have entered rural practice without those programs being in place [14, 17].

Physician Assistants and Nurse Practitioners (NP) also fill a critical role in mitigating rural-urban health care access disparities. Both professions are the fastest growing health care professions in the US and a large share of PAs and NPs work in rural health care [18–20]. Just like for physicians, however, there are also rural-urban disparities job selection for PAs and NPs [21, 22]. While prior research suggests physicians, PAs, and NPs evaluate similar aspects when choosing a job, PAs and NPs also have to evaluate state-level policies, such as scope of practice, which differentially impact their clinical responsibilities when compared to physicians [23, 24].

Our previous work studying practicing physicians, PAs, and NPs found that job, community, and personal-We next proceeded to conduct a cross-sectional observational study to examine the importance of particular characteristics regarding job choices of currently practicing physicians, PAs, and NPs as well as PA student at one of five PA training institutions in Appalachia [23, 24]. Participants rated 14 job-, community-, and personal-related characteristics with respect to how important each was for choosing their current practice location using a 5-point Likert-type answer scale (1 = Very unimportant to 5 = Very important). We received usable data from 134 providers (24.4% response rate) and 39 students (44.3% response rate) and found that the most important job-related characteristic was practice specialty for providers while students chose work hours [23, 24]. The findings from this study strongly suggested that a stated-preferences i.e., discrete choice experiment (DCE)-based approach is needed to characterize preferences and tradeoffs underlying location decisions of the healthcare provider workforce.

**Fig 2:**
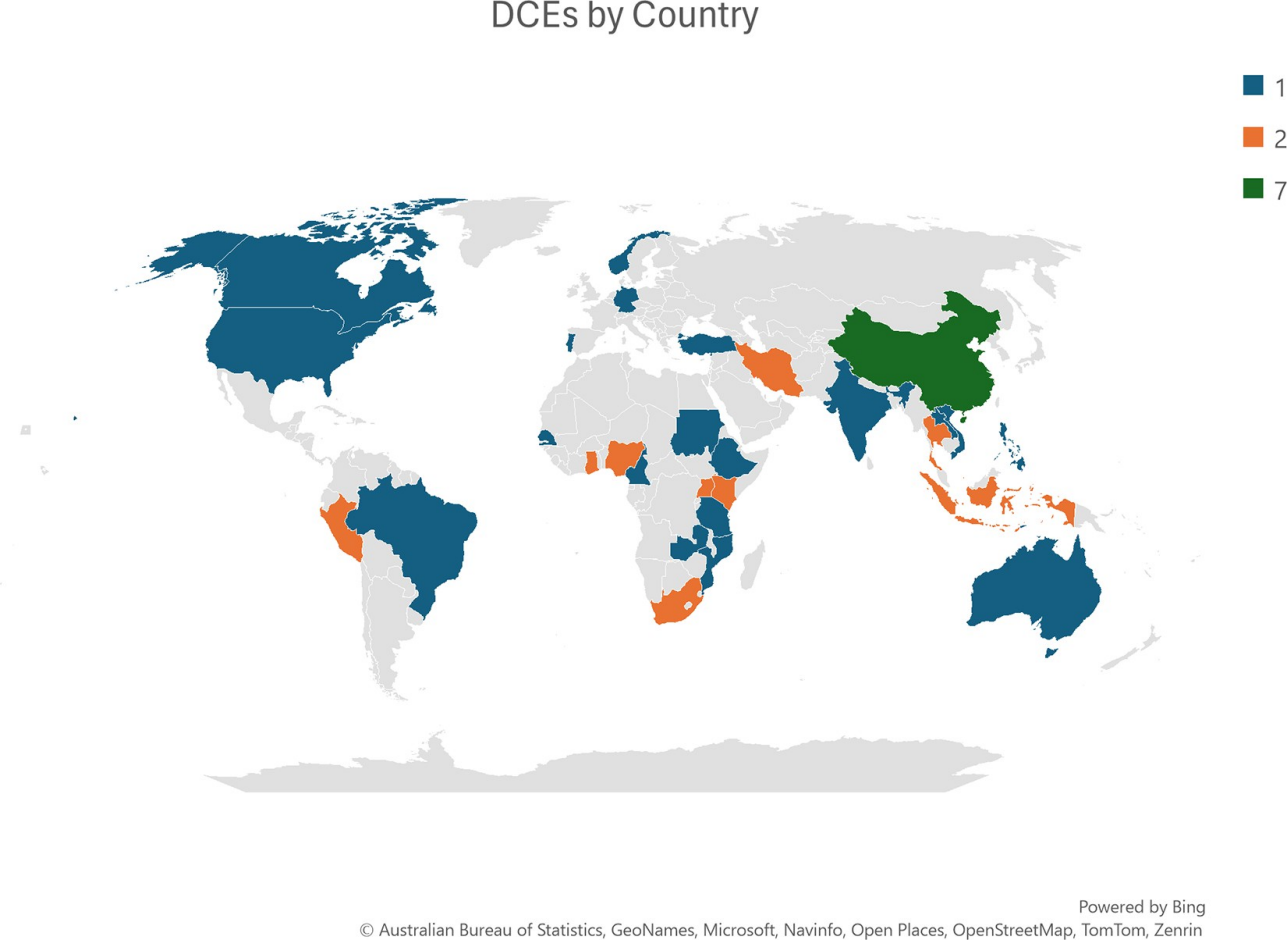
DCEs by country.

Our team is also conducting a scoping review to examine attributes and attribute levels that other studies have used to study the rural-urban access disparity (PROSPERO: CRD42023490686). Data extraction for this review is ongoing. We identified 43 articles studying almost 28 000 physicians, nurses, and health professional trainees in 30 countries (Fig 2). The findings of this scoping review regarding the attributes and attribute levels will help inform the attributes and attribute levels in a soon-to-be conducted DCE to ensure that the relevant characteristics of decision-relevant features are captured and align with previously published literature in this topic.

There has been little advancement at understanding ways to recruit and retain physicians and other health professionals to rural areas even though it has been studied intensively for decades [8]. The research to-date has failed to illuminate the processes underlying clinicians’ job choices and therefore has limited value for the prospective design of policies. New approaches to mitigating rural-urban health care access disparities are urgently needed. To identify effective policy options for recruiting clinicians to rural jobs, it is critical to develop an understanding of the complex decisions and competing factors that they evaluate and the underlying tradeoffs, indicating the need for a preference-based decision analysis. This protocol manuscript describes our approach for a rigorous, theoretically-based methodology to investigate which features physicians and non-physician clinicians value when deciding on practice locations, the relative importance of these features, and underlying tradeoffs made when selecting a job. This study is timely because, according to the American Medical Association (AMA) a plurality of physicians are employees instead of employers as of 2024, meaning that better understanding the provider job selection process is essential to improving access to care [25]. The study has three specific aims:

**Aim 1**: Identify which job-, community-, and individual-related characteristics most influence the job choices of medical residents, fellows, and PA and NP students.

**Aim 2**: Develop and conduct a DCE survey to elicit the preferences of medical residents, fellows, and PA and NP students for job-, community-, and individual-related attributes, including rural practice location.

**Aim 3**: Estimate the preferences for job-, community-, and individual-related characteristics and identify desirable rural job configuration for each group of clinicians.

### Study design and methods

This study will be the first to apply established discrete choice experiment (DCE) methodology to the context of access to care in the US. The DCE methodology is a rigorous quantitative approach to eliciting preferences. Stated preference approaches, including DCEs, have been widely used to quantify preferences for health care interventions and to inform access to health care in various high-, middle-, and low-income countries [26, 27]. Discrete choice experiments have been conducted in numerous pharmacoeconomic, health policy, and consumer preference evaluations in the US and internationally [28]. DCEs are endorsed by the World Health Organization (WHO) as a key method to identify a mix of policies and incentives to recruit health workers to rural areas [29, 30].

The DCE methodology is based on two theoretical foundations: (1) Lancaster’s consumer theory (LCT) and (2) random utility theory (RUT) [28]. The assumption behind LCT is that a person will choose a certain good or service based on an analysis of all its intrinsic characteristics, or attributes. The combination of attributes gives rise to utility, or value. RUT states that utility (value) is a latent variable that exists but cannot be directly observed. Instead, it is assumed that when an individual chooses a good or service, its utility, i.e., the value derived from the combination of all of its attributes, matches or exceeds that of all alternatives considered.

In the context of choosing a clinical job, a DCE involves repeated choices (“choice tasks”) between alternative job profiles (alternatives); each job profile is characterized by preference-relevant attributes. Attributes can include a variety of job-, community-, and personal-related characteristics [27]. This methodology provides estimates of the relative importance of these attributes when selecting a job and the tradeoffs participants make when choosing features. Discrete choice experiments are an innovative step away from characterizing the “right person” that has been the predominant focus, toward characterizing the “right job”, offering both a novel perspective and novel policy options for reducing access to care disparities for rural populations. DCEs also offer a novel approach for evaluating how job- and community-related characteristics interact with individual characteristics to allow for the design of targeted policies, a key area that has a dearth of evidence. In addition to identifying targeted policies and incentives that might increase rural job uptake, DCEs also allow for latent class analysis, which would involve identifying heterogeneous subgroups within populations that share certain preferences for jobs.

The DCE methodology can prospectively evaluate the effectiveness of targeted policies and programs prior to implementation. This “predictive” approach allows for the design and implementation of prospectively optimized policies and programs, instead of iterative trial-and-error implementations and post-hoc adaptations of failed programs. Estimates from our study can inform the design of policies for physicians, PAs, and NPs to increase uptake of rural positions and allow for direct comparisons of the potential effectiveness of new characteristics and incentives vs. established programs (e.g., debt relief). Thus, the application of a DCE to inform rural job uptake is a significant methodological advance compared to previous qualitative studies or other types of cross-sectional surveys.

### Study setting and participants

**Fig 3:**
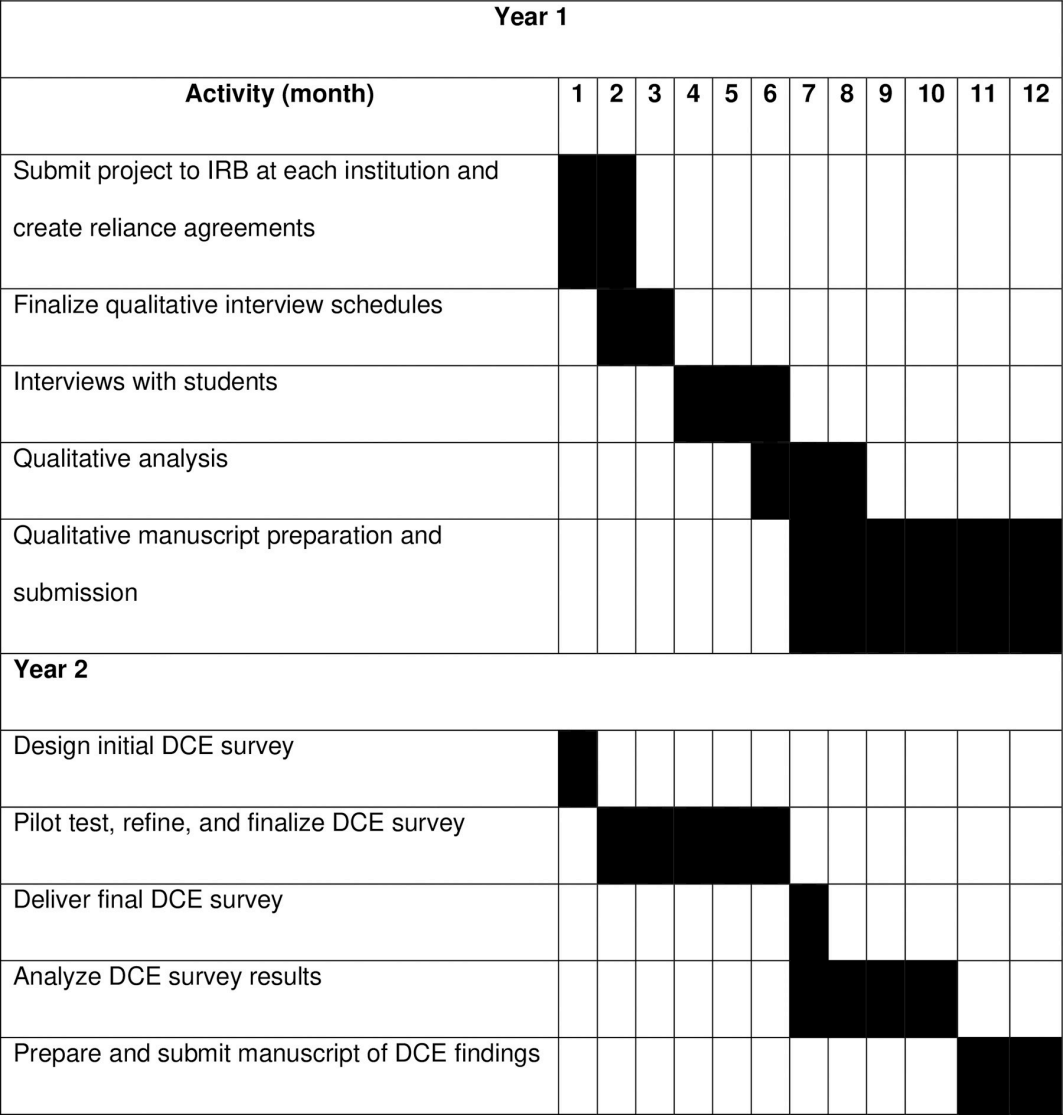
Study timeline.

The study will be conducted in multiple phases to address the specific aims and are described in detail below. Fig 3 presents the estimated timeline of the study. We have partnered with 7 medical schools across 5 US states and a territory that are within Appalachia or Appalachia-adjacent. All schools include post-graduate medical residencies and subspecialty fellowships as well as entry-level PA programs and NP programs. We also describe the subject recruitment approach for each phase below. The study will be implemented with support from the offices of rural health (physicians) and entry-level training programs for PAs and NPs) that are affiliated with each institution. We chose final-year trainees to maximize realism and because they are currently searching or have recently completed their job search.

### Outcome measure

The main outcome of interest is the relative importance of each attribute level that impacts job choice. Secondary outcome measures will be tradeoffs which will be described in the form of marginal rates of substitution (mRS). Because rural practice location will be included as an attribute, results can be used to estimate both relative preferences and probabilities of uptake of a rural job for all feasible job- and policy configurations. Specifically, all feasible job- and policy configurations can be ranked according to their expected uptake, providing concrete policy guidance on the expected effectiveness of potential policy and incentive scenarios.

### Phase 1: (ongoing) Individual in-depth interviews (IDI)

#### IDI methods

Qualitative formative work is considered good practice when designing DCEs [31]. The goal of qualitative research in DCE development is to inform attribute development. The goal of the IDIs is to (a) identify additional attributes that were not in our previous research, (b) identify levels of these attributes that represent feasible policy levers and/or tradeoffs in job decisions, and (c) prioritize the list of attributes for inclusion in the final DCE. For a successful DCE, all decision-relevant attributes must be considered and a thorough understanding of the major determinants of job choices must be developed. IDIs will help the research team identify which job-, community-, and individual-related attributes are most likely to influence practice location decisions. Importantly, we are not defining “rural” for participants *in this phase*, rather we are allowing participants to self-define “rural”. The process for developing DCEs will follow that outlined by the International Society for Pharmacoeconomics and Outcomes Research (ISPOR) Good Research Practices for Conjoint Analysis Task Force [31, 32].

To help narrow what is expected to be a large set of potentially preference-relevant attributes, we will use a variety of methods to prioritize attributes for inclusion into the DCE in the IDIs. We will also use ranking exercises, individual tradeoffs with varying feature levels, and a variation of Adaptive Choice-Based Conjoint (ACBC) exercise where respondents are asked to “design” their ideal job configuration and where tradeoffs are identified by exploring similar alternatives [31].

#### Participant sampling and recruitment

We will recruit up to 40 medical residents and fellows, and final year-PA and NP students (10 per subgroup) in Kentucky, western North Carolina, southern Ohio, and West Virginia to participate in the IDIs. We have partnered with five training institutions who will help the research team identify potentially interested participants and schedule IDIs. Evidence suggests that data saturation should occur within 9–12 interviews per group [33, 34]. Recruitment for this phase is planned between June 2024 through January 2025.

#### Data collection

The semi-structured interviews will be conducted by experienced qualitative researchers. Interviews will be conducted virtually and audio-recorded. The audio-recordings will be transcribed and uploaded into Atlas.ti to facilitate analysis.

#### Data analysis

Thematic analysis will be conducted via an iterative process that utilizes four interrelated steps: reading; coding; data display; and data reduction [35, 36]. The team will use a codebook made of a priori, structural codes based on the interview guide. At the end of Phase 2 we will have identified and prioritized key characteristics influencing the job choices of medical residents, fellows, and PA and NP students for inclusion in the DCE.

### Phase 2: DCE development, piloting, and fielding

#### Experimental design and choice set construction

**Fig 4:**
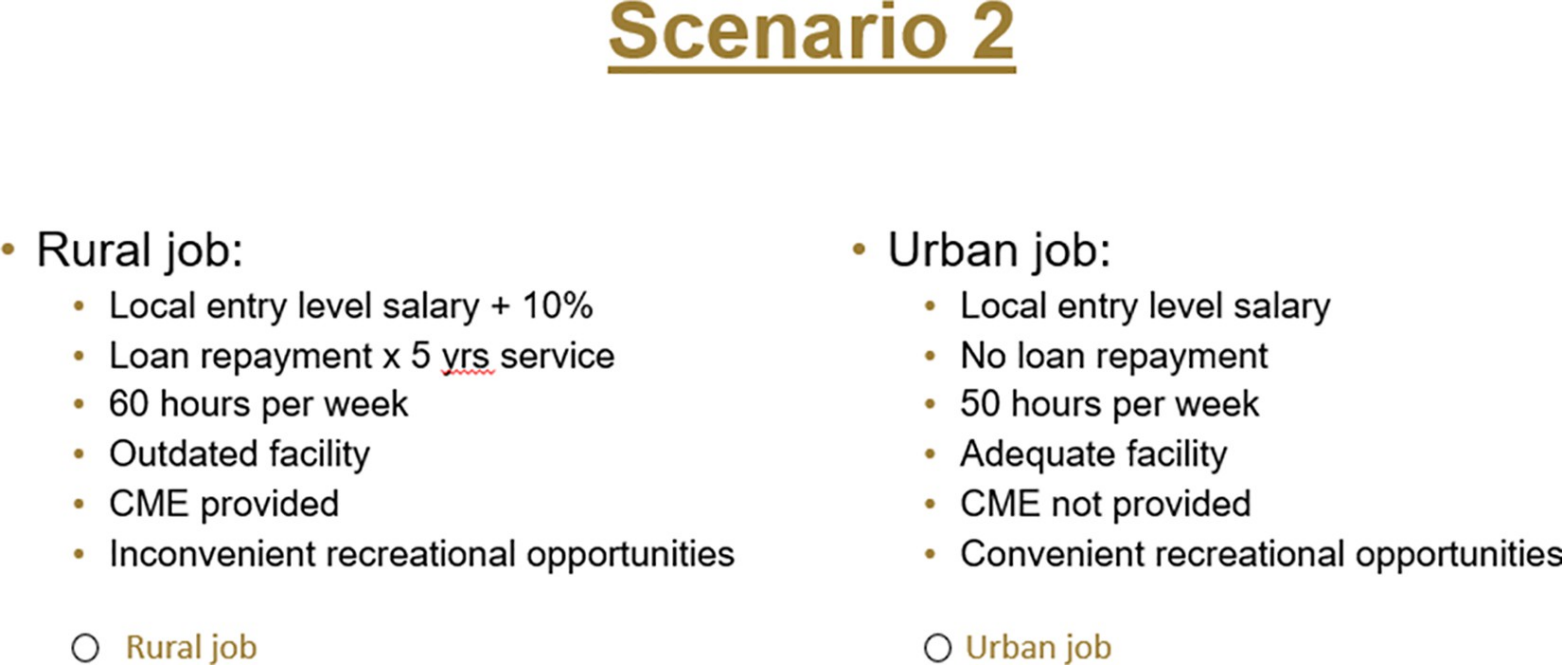
Example DCE choice task.

The DCE choice tasks will resemble the task shown in (Fig 4). Columns represent job options. Rows describe the attributes identified in Phase 2. Cells represent feasible levels for each attribute. DCE attributes are typically represented by 2–4 levels, which can include nominal, ordinal, or cardinal values. Rural practice location will be one attribute level of interest. Each respondent will be asked to complete 10–15 choice tasks. Each choice task will involve the comparison of two hypothetical job options. The DCE will also include an option of taking neither job (‘opt-out’), which reflects a realistic view of the provider job market. This opt-out option provides a reference point that allows for estimates of the potential uptake of job configurations of interest [37].

#### Questionnaire design

Robust statistical results can be obtained from a fractional factorial design of several dozen questions, implemented in a few different versions of the survey [32]. Formally, we will use Sawtooth software (Provo, UT) to identify a *d*-efficient, partial factorial design that has good attribute level balance to ensure that each respondent will see most or all attribute levels. Factorial randomized designs allow for researchers to assess interactions among the studied factors, which is not possible with other types of randomized trials. To incorporate preference heterogeneity into the statistical design, the design will be optimized for mixed multinomial logit analysis, with simulations drawing priors from the estimated parameter distributions.

In addition to the DCE section of the survey, participants will also complete items to assess potential individual-level correlates of preferences. The survey will assess socio-demographic characteristics (e.g., age, gender identity), background, student loans, specialty training, rotations in rural areas, family constraints, and other correlates.

Prior to fielding, the final survey will be pre-tested and iteratively revised using up to 20 cognitive interviews [34]. Participants will be asked to “think aloud” while completing the DCE survey; interviewers will evaluate understanding and probe why selected answers were chosen. Sawtooth’s choice-based conjoint software will be used to design host the final DCE. A pilot study with 30 purposively selected persons will estimate priors to inform the statistical design.

#### Sample size and statistical power

Based on our past experience and DCE development guidelines, which combined the number of respondents (n), choice tasks, (t = 10–15), alternatives per task (a = 3) and the maximum number of feature levels (c = 4) and established a cutoff of nta/c ≥ 500, we calculated that a sample size of n = 100 per cohort will provide sufficient power to characterize group-specific mean preferences separately for residents, fellows, and PA and NP students or for each partner institution, while the combined sample size will allow for the analysis of heterogeneity using a latent class model (see DCE data analysis below) [38].

#### Participant sampling

We will sample medical residents, fellows, and final-year PA and NP students at comparable times in their training (e.g., approximately 6 months from program completion). We will distribute the DCE survey to 400 medical residents, fellows, and final-year PA and NP students with selected university partners as stated above, with a goal to recruit at least 100 participants per professional group. One individual at each program will be responsible for ensuring survey distribution via email distribution lists and sending reminder emails for non-respondents. Each trainee cohort will complete the survey at comparable times in their training, i.e., approximately 6 months prior to program end or graduation. Recruitment for this phase is scheduled between April and June 2025.

### DCE data analysis

We will analyze the choice data using mixed (random parameter) logit models [32]. The binary dependent variable will be 1 for the most preferred of the 3 alternatives (including the opt-out alternative) and 0 for the other two alternatives in each choice task. The independent variables will be the effects-coded levels of each attribute. The mixed logit specification will be estimated in STATA 16 (StataCorp, College Station, TX) allows for variation in preferences across respondents as well as error correlation across choice tasks within respondents. Separate models will be estimated for each subgroup (residents, fellows, and PA and NP students). Generalized Hausman tests will evaluate whether preference estimates differ between groups. We will also test latest-class models on the pooled data, which may provide a better fit if the sample breaks down statistically into a few discrete classes. Latent class models will be estimated using Latent Gold Choice 5.0 software.

The preference weights estimated using group-specific mixed logit models are the primary outcome of Phase 4. Positive parameter estimates for effects-coded attribute levels indicate that an attribute level is more preferred compared to the mean, while negative parameter estimates indicate aversion. Tradeoffs are described in the form of mRS. Because rural practice location will be included as an attribute level, results can be used to estimate both relative preferences and probabilities of uptake of a rural job for all feasible job- and policy configurations. Most importantly, all feasible combinations of job-, community-, and individual-related characteristics can be ranked according to their expected uptake, providing concrete policy guidance on the expected effectiveness of potential policy scenarios.

#### Data security and confidentiality

A research data security plan has been developed to ensure that data are kept in compliance with relevant privacy regulations. All surveys will be confidential and will not contain any identifying information. Regarding the qualitative formative work, all participant references to specific people and places will be replaced with “xx” to ensure anonymity. Data will be transferred through the use of the HIPAA compliant Box cloud software.

#### Ethics concerns

This study has been approved by the Wake Forest University School of Medicine Institutional Review Board. All phases of the research study that involve human participants will be conducted according to the Declaration of Helsinki and the Belmont Report. All identities of the participants’ survey and IDI data will be blinded to the research team to ensure anonymity.

## Discussion

Deciding on a job is a complex process where providers weigh and trade between multiple factors and compare job offers to decide on which is better for themselves and often, their family. In order to accomplish the study’s objective of identifying an optimal set of policy options and incentives that would attract clinicians to rural Appalachia, understanding this complex decision-making process is essential. This DCE will be the first to study preference-relevant features with a goal to improve the rural-urban access disparity in the United States. This study is timely because the majority of currently trained physicians will become employees instead of employers [25]. We chose to focus on rural Appalachia because this area is well-known for being mostly rural and for having some of the worst health outcomes in the country [4, 5]. The optimal set of policy options will be clearly communicated to regional stakeholders using policy briefs and meetings. This DCE will advance what is known about the rural-urban because it will be the first to focus on actionable characteristics that can directly inform policy. The DCE methodology can prospectively evaluate the effectiveness of targeted policies and programs prior to implementation, which can save money and time compared to iterative trial-and-error implementations and post-hoc adaptations of failed programs. Estimates from our study can directly inform policy because we are focusing on actionable characteristics that can be modified to allow for direct comparisons of the potential effectiveness of new approaches versus established programs. The findings of this study can also be used to design pragmatic trials that compare the effectiveness of different policy options and incentives. In conclusion, this study will make scientific and methodological contributions to US health care delivery science as well as has the potential to make a significant public health impact for rural Americans.

## Supporting information

S1 File (PDF)
