# Supplementary material for: A discrete choice experiment with health professions trainees to improve the urban-rural health care access disparity in Appalachia: Study protocol
Source: PLoS One. 2025 Jan 13;20(1):e0316521. doi: 10.1371/journal.pone.0316521 (PMC11729937; doi:10.1371/journal.pone.0316521)
Supplement: S1 File — (PDF) [file pone.0316521.s001.pdf]

SUMMARY STATEMENT

PROGRAM CONTACT: (Privileged Communication) *Release Date:* 07/23/2023

Revised Date:

Application Number: Principal Investigators (Listed Alphabetically): 1R21MD019127-01

(Contact)

Applicant Organization: WAKE FOREST UNIVERSITY HEALTH SCIENCES

Review Group: HHD
Healthcare and Health Disparities Study Section

Meeting Date: 06/22/2023
Council: OCT 2023
Requested Start: 12/01/2023

Opportunity Number: PAR-20-150
PCC: CPS07

Dual IC(s): LM

Project Title: AHCRA: Access to Health Care in Rural Appalachia

SRG Action: Impact Score:25 Percentile:8 +
Next Steps: Visit https://grants.nih.gov/grants/next_steps.htm

Human Subjects: 48-At time of award, restrictions will apply

Animal Subjects: 10-No live vertebrate animals involved for competing appl.
Gender: 1A-Both genders, scientifically acceptable
Minority: 1A-Minorities and non-minorities, scientifically acceptable
Age: 3U-No children included, scientifically unacceptable

Project Year	Direct Costs	Estimated Requested	Total Cost
1	150,000		243,403
2	125,000		202,836
TOTAL	275,000		446,239

ADMINISTRATIVE BUDGET NOTE: The budget shown is the requested budget and has not been adjusted to reflect any recommendations made by reviewers. If an award is planned, the costs will be calculated by Institute grants management staff based on the recommendations outlined below in the COMMITTEE BUDGET RECOMMENDATIONS section.

1R21MD019127-01

INCLUSION ACROSS THE LIFESPAN PLAN UNACCEPTABLE
PROTECTION OF HUMAN SUBJECTS UNACCEPTABLE

RESUME AND SUMMARY OF DISCUSSION: The proposed research seeks to evaluate a theoretically grounded, quantitative experimental approach to studying reasons underlying the maldistribution in the US healthcare clinician workforce, and to identify potential policy approaches to address these disparities. The rigor of the prior research is very strong, and the project's significance lies in its ability to advance what is currently known about policy options that will address rural-urban access to care disparities. This is a highly successful investigative team that is well-experienced, compatible, and has the necessary expertise to ensure the success of the project. The panel was confident that the aims and objectives of the study could be achieved given the composition of the team. The panel was also enthusiastic about many strengths of the study including the team's experience practicing in rural areas, the novel focus of job characteristics, the rigor of the design and methods, and team's ability to leverage preliminary studies to support the current work. Some of the weaknesses that were mentioned during the discussion include Aim 1 seems redundant, the timeline is ambitious, the recruitment strategy is missing details and may not feasible, and the sample size may not aide in detecting differences. Nonetheless, the noted weaknesses can be addressed and did not significantly diminish the strengths and the significance of the study. If implemented as planned, the project is expected to have a high overall impact on the field.

DESCRIPTION (provided by applicant): Approximately one-fifth of the United States (US) population lives in rural areas. Americans in rural areas often experience poorer health and engage in fewer health promotion activities compared to their more urban counterparts. Currently there are more healthcare clinicians per capita in urban areas compared to rural areas in the US. Despite a greater need for healthcare in rural areas, the US experiences a paradox regarding access to care in rural areas: Americans with the greatest need for healthcare experience the least access to care. While this clinician maldistribution has been present for decades, relatively little is known about why clinicians choose to locate in a specific area. Even more concerning is that the clinician maldistribution is worsening over time, forcing millions of Americans to drive farther to obtain healthcare. The disparity in access to care increases both morbidity, mortality, and increases health care costs. We propose to evaluate a theoretically-grounded, quantitative experimental approach to studying reasons underlying the maldistribution in the US healthcare clinician workforce, and to identify potential policy approaches to address these disparities. Specifically, we will evaluate the relative importance of job-, community-, and personal-related attributes for medical residents and fellows as well as physician assistant (PA) and nurse practitioner (NP) students using a discrete choice experiment (DCE). The DCE methodology is a rigorous survey method used to elicit preferences, quantify tradeoffs, and understand decisionmaking. This study is innovative because it will be the first to use DCE methods to identify policy options for increasing the attractiveness of rural jobs in the US. Our proposed study will address three aims: Aim 1 will elicit individual-, community-, and job-related preferences that influence decisions on whether to choose a clinical position from residents and fellows, as well as PA and NP students. Aim 2 will develop, pilot-test, and deliver the DCE survey to characterize the most important attributes and their levels that influence whether to choose a particular clinical position. Aim 3 will estimate the relative importance of the most important attributes and their levels that influence whether a clinician chooses a particular clinical position. This proposed research will provide policy- and decision-makers with an important first step towards designing policy options and incentives that increase the attractiveness of healthcare jobs in rural areas. More broadly, this proposed research will also provide our research team pilot data from which to generate future research with healthcare clinicians and identifying policy options and incentives to retain them in rural areas.

PUBLIC HEALTH RELEVANCE: The United States is experiencing a healthcare clinician maldistribution that negatively affects access to care and health outcomes for millions of Americans. The rural-urban access to care disparity is a well-known but growing public health threat for rural

Americans. The findings of this study will identify an optimal set of policy options and incentives that would attract clinicians to a rural area.

CRITIQUE 1

Significance: 2
Investigator(s): 1
Innovation: 1
Approach: 2
Environment: 1

Overall Impact: Rigorous prior research has established that rural residents experience greater morbidity and mortality than their suburban and urban counterparts. This is in large part due to the strong and increasing preference for clinical care providers to live in urban and suburban locations. This preference has created a long-standing rural-urban access to care disparity. This application seeks to apply a novel method for better understanding care provider decision making when it comes to selecting a job assignment with the goals to identify most preferable rural job configurations that can be translated into policies to best attract and ultimately retain a rural living clinical work force. Strengths of the application include: a novel paradigm shift from characterizing the rural-urban access to care disparity as “finding the right person” – namely clinicians from rural areas who are themselves growing fewer – to “finding the right job” – to attract a more variable clinician demographic. The research team is strong and has all of the needed training and expertise to conduct the study. Preliminary studies have proven the potential and feasibility of applying the decision choice experiment model to the context of health care clinicians’ job choices and the proposed study would further expand the prior work to elucidate optimal rural job characteristics that best predict rural job preference selection by clinicians who are coming out of training and/or residency. The proposed formative and qualitative methods for Aim1 are rigorous and the DCE development, pilot, and testing for Aim 2 will ensure a valid measure. Tests for Aim 3 appear to be well powered. There is a dissemination plan that includes translation of findings to policy makers. Only few and minor weaknesses were identified with some concern with the modifiability of the characteristics identified by the DCE and their real-world translation into policy and in the generalizability of findings to other very low resource regions such as Alaska, but the method is flexible enough to be adaptable to identifying region specific characteristics as well. Overall, this application has the potential to have a high impact for a highly under-resourced region of our nation, Appalachia region, and should be done and can be done by the competent and experienced team leading the study.

1. Significance:

Strengths

- Addresses the acute rural-urban health care access disparity utilizing a novel application of an established decision choice experiments methodology to best understand health care clinicians’ job choices.
- Importantly considers primary care and subspeciality physicians’ preferences as well as PAs and NPs and adds in clinician specific preferences such as considering state-level policies for PAs and NPs to best measure autonomy in practice.
- The study team includes practicing PAs who work in rural areas to best inform the characteristics that comprise the quantifiable DCE.

- The study focuses in one predominately rural region, Appalachia region, with among the highest rural-urban health care access disparities and health inequities in the nation.

Weaknesses

- Some minor concern with generalizability of health care clinician's job preferences to other rural regions of the country, in particular in Alaska where access to care disparities are the very highest and health inequities most acute.

2. Investigator(s):

Strengths

- The PI ( ) is a trained pharmacoepidemiologist with expertise is the discrete choice experiments methodology proposed in the current study aims.
- The PI has a strong publication record and has led prior research studies.
- The Co-PI ( ) is contact PI for two R01s and an R21 and has a strong background in health disparities research including rural-urban disparities with expertise in qualitative and discrete choice experiments methodologies.
- Co-PIs ( ) both provide a clinical PA professional perspective to the study team, and both have experience working in rural areas.
- The team has collaborated and co-authored publications.

Weaknesses

- None noted by reviewer.

3. Innovation:

Strengths

- First study to apply DCE to the context of access to care in the US.
- DCE is a novel approach for informing access to health care policy at a state and national level.
- Policy level interventions are also under-utilized in this space with a prior focus being in "finding the right person" to fill rural positions rather than on larger scale promotion of "right jobs" with broader appeal to health care clinicians of various types.

Weaknesses

- None noted by reviewer.

4. Approach:

Strengths

- The team successfully collaborated on a pilot study that surveyed healthcare clinicians (n=130 physicians, PAs, and NPs) and PA students (n=43) to assess the importance of diverse job-, community-, and individual-related attributes that had been previously hypothesized to influence

  job choices. The pilot study confirmed the importance of all of the factors initially identified but did not elucidate preferences and rankings of importance for each of the characteristics. The design of the proposed study will utilize DCE to formulate predictive preference models for rural choice making.

- Study region connected to the Appalachian Translational Research Network is well reasoned and the team has experience engaging this network in studies.
- The qualitative formative Aim 1 is well-reasoned, and methods are rigorous.
- Aim 3 is sufficiently powered and considerations of limitations including realism and the realwork fit between DCEs and actual behavior and choice has been shown in prior work to be matched.

Weaknesses

- The Aim 1 formative work might be somewhat duplicative of the preliminary pilot work. Do the investigators anticipate new themes emerging from the 30 interviews that are qualitatively and quantifiable different from the characteristics in the pilot survey?
- Aim 2 may be overly ambitious in scope and procedures for the purposes of this study. 20 cognitive interviews at pre-test is a large number. The pilot of 30 surveys may be sufficient.

5. Environment:

Strengths

- Wake Forest University and Medical School provide all needed academic, intellectual, and administrative supports for the proposed research.
- There is a ready recruitment pool with Wake Forest School of Medicine for the purposes of the study.

Weaknesses

- Resources for the co-PI ( ) and Co-I ( ) are not listed.

Protections for Human Subjects:

Data and Safety Monitoring Plan (Applicable for Clinical Trials Only):

Inclusion Plans:

- Sex/Gender: Distribution justified scientifically
- Race/Ethnicity: Distribution justified scientifically
- For NIH-Defined Phase III trials, Plans for valid design and analysis: Not applicable
- Inclusion/Exclusion Based on Age: Distribution not justified scientifically

Resource Sharing Plans:

Acceptable

Budget and Period of Support:

Recommend as Requested

**CRITIQUE**	3
Significance:	3
Investigator(s): 1Innovation:	3
Approach:	3

Environment: 1

Overall Impact: This is a project to examine the reasons underlying the maldistribution in US Healthcare clinician work force with less and decreasing location of clinicians to rural areas. This includes physician, PA and NP. Uses Discrete choice experiment methodology. The rigor of prior research is good. Score driving strengths were the careful construction of the instrument and the design of the study. Weakness mostly related to the potential lack of generalizability and a ambitious recruiting timeline for participants.

1. Significance:

Strengths

- The disparity in access to care in rural US is important, with less clinicians in rural areas. This problem is worsening.
- The methods proposed are robust
- The focus on Appalachia should allow clearer delineation of potential solutions while limiting generalizability.
- The study team includes both physicians and physician assistant. **Weaknesses**
- There is a lack of detail in the sample selection and no mention of effect sizes

2. Investigator(s):

Strengths

- MPI has a PhD in Pharmaceutical Sciences and is an Associate Professor in Public Health Sciences and Department of PA Studies
- MPI has a PHD in Health Policy and Administration and is an Associate Professor in Public Health. He has experience, publications and prior funding in the use and analysis of discreet choice experiments

Weaknesses

- None noted by reviewer.

3. Innovation:

Strengths

- Discreet choice methodology examining the effect of job, community, and individual characteristics

The institutions whose students are being surveyed are all Appalachian located with strong rural health care components.

- The emphasis on creating the right job rather than finding the right person is innovative.

Weaknesses

- None noted by reviewer.

4. Approach:

Strengths

- Discreet choice methodology in quantifying community, job and individual characteristics driving practice choice.
- Fractional factorial design allows for efficient design with generalizable to sample
- Appropriate preliminary/pilot data.
- The concentration on Appalachia will allow the investigators to clearly understand the issues in a discrete area of the country.

Weaknesses

- It is not clear if the results will be generalizable to different schools or a different group of students. Since the students will be volunteers the results may not be generalizable
- The sample size does not give any detectable effect sizes.
- The recruitment of participants seems very ambitious.
- Sex as a biologic variable was not addressed.

5. Environment:

Strengths

- Strong and supportive environment.

Weaknesses

- None noted by reviewer.

Protections for Human Subjects:

Unacceptable Risks and/or Inadequate Protections

- There needs more detail in how participants are recruited. The numbers in the enrollment table don't match each other or the proposal when the pilot and cognitive interviewing are considered.

Data and Safety Monitoring Plan (Applicable for Clinical Trials Only):

Not Applicable (No Clinical Trials)

Inclusion Plans:

- Sex/Gender: Distribution justified scientifically
- Race/Ethnicity: Distribution justified scientifically

  For NIH-Defined Phase III trials, Plans for valid design and analysis: Not applicable
- Inclusion/Exclusion Based on Age: Distribution justified scientifically

Resource Sharing Plans:

- No resource sharing plan given

Budget and Period of Support:

Recommend as Requested

CRITIQUE 3

Significance: 3
Investigator(s): 2
Innovation: 3
Approach: 4
Environment: 2

Overall Impact: This study proposes to use a cross-sectional design to identify factors underlying clinician's job choices for jobs in rural areas. The study assesses job preferences for PAs, NPs, and physicians in NC, WV, OH and KY. The focus on rural area disparities is significant. Other strengths include a strong investigative team, preliminary work in this area, and partnership with 4 rural health offices that are members of the Appalachian Translational Research Network. Moderate weaknesses include inclusion of students from only 4 states and selection bias in who responds, lack of attention to intersectionality in recruitment; low innovation for Aim 1 and concerns around enrolling 400 health care providers for Aim 2. The study seems rather ambitious for the proposed timeline. Overall, it's likely to have high to medium impact.

1. Significance:

Strengths

- Those who live in rural areas face greater morbidity and mortality and this project focuses on an important problem of clinician supply in rural areas
- Access to health care needs will remain limited for patients living in rural areas.
- Focus on better understanding factors that would incentivize current and future clinicians to choose to work in rural locations and remain there

Weaknesses

- Inclusion of students from only 4 states and selection bias in who responds to this study •

  Lack of consideration of intersectionality in terms of recruitment into Aims 1 and 2

2. Investigator(s):

Strengths

mPI Dr. (health services researcher) and Dr. (expert in the use of DCE methods) bring unique expertise to the project

- Co-I serves as an expert on rural health
- Dr. is a physician in a rural area and an Assistant Professor of Family Medicine at Atrium Health
- Dr. is an expert in access to care and the healthcare workforce
- Partnership with 4 offices of rural health

Weaknesses

- None noted by reviewer.

3. Innovation: Strengths

- Application of discrete choice experiments to the context of clinician’s jobs

Weaknesses

- Lack of attention to diversity in recruitment and implications of these results

4. Approach: Strengths

- Strong preliminary data
- Study population includes medical residents, fellows, and physician assistant (PA) and nurse practitioner (NP) students who have trained in the Appalachian region
- Use of discrete choice models **Weaknesses**
- Aim 1 seems really formative when the proposal already discusses existing analyses by the team that can speak to the different factors that influence job choices
- No discussion of alternative solutions if “additional themes and attributes not identified in previous research” do not emerge from Aim 1
- Sample for Aim 1 is rather small (10 per group) to generate a comprehensive list of key factors and doesn’t seem to consider intersectionality
- Concerns around enrollment of 400 participants for Aim 2
- Concerns around latent class models for Aim 3 to determine relative importance of factors
- Bias from self-selection for those who participate concerns around generalizability of findings and how some of these factors are modifiable toward actually impacting policy

5. Environment:

Strengths

- Wake Forest University Health Sciences has adequate resources for this work

Weaknesses

None noted by reviewer.

Study Timeline:

Strengths

- None noted by reviewer.

Weaknesses

- Timeline seems inadequate for the proposed analyses.

Protections for Human Subjects:

Acceptable Risks and/or Adequate Protections

Data and Safety Monitoring Plan (Applicable for Clinical Trials Only):

Not Applicable (No Clinical Trials)

Inclusion Plans:

- Sex/Gender: Distribution justified scientifically
- Race/Ethnicity: Distribution not justified scientifically
- For NIH-Defined Phase III trials, Plans for valid design and analysis: Not applicable
- Inclusion/Exclusion Based on Age: Distribution not justified scientifically
- Not clear why older adults and those of retirement age are excluded

Resource Sharing Plans:

Acceptable

Budget and Period of Support:

Recommend as Requested

THE FOLLOWING SECTIONS WERE PREPARED BY THE SCIENTIFIC REVIEW OFFICER TO SUMMARIZE THE OUTCOME OF DISCUSSIONS OF THE REVIEW COMMITTEE, OR REVIEWERS' WRITTEN CRITIQUES, ON THE FOLLOWING ISSUES:

PROTECTION OF HUMAN SUBJECTS: UNACCEPTABLE

This application includes potential risks to the protections of human subjects that should be addressed as recommended by reviewers.

INCLUSION OF WOMEN PLAN: ACCEPTABLE

INCLUSION OF MINORITIES PLAN: ACCEPTABLE

INCLUSION ACROSS THE LIFESPAN PLAN: UNACCEPTABLE

This information is not sufficiently described in the application, especially as it relates to the exclusion criteria.

COMMITTEE BUDGET RECOMMENDATIONS: The budget was recommended as requested.

Footnotes for 1R21MD019127-01; PI Name:

+ Derived from the range of percentile values calculated for the study section that reviewed this application.

NIH has modified its policy regarding the receipt of resubmissions (amended applications).See Guide Notice NOT-OD-18-197 at https://grants.nih.gov/grants/guide/notice-files/NOT-OD-18197.html. The impact/priority score is calculated after discussion of an application by averaging the overall scores (1-9) given by all voting reviewers on the committee and multiplying by 10. The criterion scores are submitted prior to the meeting by the individual reviewers assigned to an application, and are not discussed specifically at the review meeting or calculated into the overall impact score. Some applications also receive a percentile ranking. For details on the review process, see http://grants.nih.gov/grants/peer_review_process.htm#scoring.

MEETING ROSTER

Healthcare and Health Disparities Study Section
Healthcare Delivery and Methodologies Integrated Review Group
CENTER FOR SCIENTIFIC REVIEW
HHD
06/22/2023 - 06/23/2023

CHAIRPERSON(S)

PROFESSOR
DEPARTMENT OF PEDIATRICS
PERELMAN SCHOOL OF MEDICINE
UNIVERSITY OF PENNSYLVANIA
PHILADELPHIA, PA 19104

MEMBERS

ASSISTANT RESEARCH PROFESSOR
MCCOURT SCHOOL OF PUBLIC POLICY
MASSIVE DATA INSTITUTE
GEORGETOWN UNIVERSITY
WASHINGTON, DC 20016

DIVISION OF AGING AND DEMENTIA
INSTITUTE FOR PUBLIC HEALTH
SCHOOL OF MEDICINE
WASHINGTON UNIVERSITY OF ST LOUIS
ST. LOUIS, MO 63130

DEPARTMENT OF NEUROLOGY
COMPREHENSIVE CENTER OF BRAIN HEALTH
MILLER SCHOOL OF MEDICINE
UNIVERSITY OF MIAMI
MIAMI, FL 33133

ASSISTANT PROFESSOR
DEPARTMENT OF HEALTH POLICY AND MANAGEMENT
SCHOOL OF PUBLIC HEALTH AND TROPICAL MEDICINE
TULANE UNIVERSITY
NEW ORLEANS, LA 70112

ASSOCIATE PROFESSOR OF PEDIATRICS
COLUMBIA UNIVERSITY HEALTH SCIENCES
NEW YORK, NY 10027

PROFESSOR
DEPARTMENT OF PSYCHIATRY AND BEHAVIORAL SCIENCE
MEDICAL UNIVERSITY OF SOUTH CAROLINA

CHARLESTON, SC 29401

PROFESSOR
DEPARTMENT OF PEDIATRICS
UNIVERSITY OF ARKANSAS FOR MEDICAL SCIENCES LITTLE ROCK, AR 72202

ASSOCIATE PROFESSOR
ASSOCIATE DEAN OF DIVERSITY, EQUITY AND INCLUSION
PEDIATRIC INFECTIOUS DISEASES
COLLEGE OF MEDICINE
UNIVERSITY OF NEBRASKA MEDICAL CENTER
OMAHA, NE 68198

Notice of NIH Policy to All Applicants: Meeting rosters are provided for information purposes only. Applicant investigators and institutional officials must not communicate directly with study section members about an application before or after the review. Failure to observe this policy will create a serious breach of integrity in the peer review process, and may lead to actions outlined in NOT-OD-22-044 at https://grants.nih.gov/grants/guide/notice-files/NOT-OD-22-044.html, including removal of the application from immediate review.

VICE CHAIR FOR RESEARCH, RONALD O.
PERELMAN DEPARTMENT OF
EMERGENCY MEDICINE
NYU GROSSMAN SCHOOL OF
MEDICINE
NEW YORK, NY 10016

DEPARTMENT OF HEALTH AND
BIOMEDICAL SCIENCES
UNIVERSITY OF TEXAS RIO GRANDE
VALLEY EDINBURG, TX 78539

PROFESSOR
BROWN SCHOOL OF SOCIAL WORK
WASHINGTON UNIVERSITY

ST. LOUIS, MO 63130
ASSOCIATE DEAN FOR RESEARCH AND

ASSOCIATE PROFESSOR
SCHOOL OF NURSING
DUKE UNIVERSITY

DURHAM, NC 27710

ASSOCIATE PROFESSOR
DEPARTMENT OF COMMUNITY HEALTH SYSTEMS
SCHOOL OF NURSING
UNIVERSITY OF CALIFORNIA, SAN FRANCISCO
SAN FRANCISCO, CA 94143

PROFESSOR
DEPARTMENT OF SURGERY
PERELMAN CENTER FOR ADVANCED MEDICINE
PENNSYLVANIA UNIVERSITY
PHILADELPHIA, PA 19104

PROFESSOR AND ASSOCIATE CHAIR FOR RESEARCH
DEPARTMENT OF HEALTH SYSTEMS
AND POPULATION HEALTH
UNIVERSITY OF WASHINGTON
SEATTLE, WA 98195

PROFESSOR
DEPARTMENT OF HEALTH DISPARITIES RESEARCH
UNIVERSITY OF TEXAS M.D. ANDERSON CANCER CENTER HOUSTON, TX 77030

ASSOCIATE PROFESSOR
DEPARTMENT OF NEUROLOGY
SCHOOL OF MEDICINE
UNIVERSITY OF CALIFORNIA, DAVIS
SACRAMENTO, CA 95817

PROFESSOR
DEPARTMENT OF SOCIAL AND BEHAVIORAL HEALTH SCHOOL OF
MEDICINE

VIRGINIA COMMONWEALTH UNIVERSITY RICHMOND, VA
23298-0149
PROFESSOR

PROFESSOR
COLLEGE OF NURSING

SCHOOL OF NURSING
UNIVERSITY OF KANSAS

DIRECTOR, CENTER FOR ALASKA NATIVE HEALTH
RESEARCH
ASSOCIATE PROFESSOR, INSTITUTE OF ARCTIC BIOLOGY
UNIVERSITY OF ALASKA FAIRBANKS FAIRBANKS, AK 99712

ASSISTANT PROFESSOR
SANDRA ROSENBAUM SCHOOL OF SOCIAL WORK
UNIVERSITY OF WISCONSIN–MADISON
MADISON, WI 53706

PROFESSOR
SINCLAIR SCHOOL OF NURSING
UNIVERSITY OF MISSOURI COLUMBIA, MO
65211

ASSOCIATE PROFESSOR OF MEDICINE

SECTION OF GENERAL INTERNAL MEDICINE
MACLEAN CENTER FOR CLINICAL MEDICAL ETHICS
THE UNIVERSITY OF CHICAGO MEDICINE CHICAGO, IL 60637

ASSISTANT PROFESSOR

FRANCES PAYNE BOLTON SCHOOL OF NURSING
CASE WESTERN RESERVE UNIVERSITY
CLEVELAND, OH 44106

PROFESSOR

DIVISION OF HEALTH POLICY AND MANAGEMENT
UNIVERSITY OF MINNESOTA SCHOOL OF PUBLIC HEALTH
MINNEAPOLIS, MN 55455

PROFESSOR

CENTER FOR HEALTH RESEARCH
LOMA LINDA UNIVERSITY
LOMA LINDA, CA 92350

PROFESSOR
KANSAS CITY, KS 66160
UNIVERSITY OF KENTUCKY
LEXINGTON, KY 40536
HUMAN DEVELOPMENT AND FAMILY SCIENCE

NORTON SCHOOL OF HUMAN ECOLOGY
THE UNIVERSITY OF ARIZONA
TUCSON, AZ 85721

PROFESSOR
DEPARTMENT OF HEALTH POLICY AND MANAGEMENT
GILLINGS SCHOOL OF GLOBAL PUBLIC HEALTH
UNIVERSITY OF NORTH CAROLINA AT CHAPEL HILL
CHAPEL HILL, NC 27599

ASSOCIATE PROFESSOR
DEPARTMENT OF POPULATION
AND PUBLIC HEALTH SCIENCES
KECK SCHOOL OF MEDICINE
UNIVERSITY OF SOUTHERN CALIFORNIA
LOS ANGELES, CA 90033

PROFESSOR
DEPARTMENT OF GEOGRAPHY AND ANTHROPOLOGY
LOUISIANA STATE UNIVERSITY BATON ROUGE,
LA 70803

RESEARCHER
RTI INTERNATIONAL MEDIA, PA
19063

ASSOCIATE PROFESSOR
DEPARTMENT OF EPIDEMIOLOGY
BOSTON UNIVERSITY SCHOOL OF PUBLIC HEALTH
BOSTON, MA 02118

SCIENTIFIC REVIEW OFFICER

SCIENTIFIC REVIEW OFFICER
CENTER FOR SCIENTIFIC REVIEW
NATIONAL INSTITUTES OF HEALTH BETHESDA, MD
20892

EXTRAMURAL SUPPORT ASSISTANT

LEAD EXTRAMURAL SUPPORT ASSISTANT
CENTER FOR SCIENTIFIC REVIEW
NATIONAL INSTITUTES OF HEALTH
BETHESDA, MD 20817

* Temporary Member. For grant applications, temporary members may participate in the entire meeting or may review only selected applications as needed.

Consultants are required to absent themselves from the room during the review of any application if their presence would constitute or appear to constitute a conflict of interest.
